# Correlation between innate and adaptive immunity response in TB children post BCG vaccination. Is it effective or not?: Cross-sectional study

**DOI:** 10.1016/j.amsu.2022.103586

**Published:** 2022-04-06

**Authors:** Rahmini Shabariah, Farsida, Mochammad Hatta, Ilhamjaya Patellongi, Irfan Idris, Arif Santoso, Andi Asadul Islam, Rosdiana Natzir, Bob Wahyudin, Warsinggih, Sanjoyo

**Affiliations:** a Dept of Pediatric, Faculty of Medicine and Health, Universitas Muhammadiyah Jakarta; b Dept of Community Medicine, Faculty of Medicine and Health, Universitas Muhammadiyah Jakarta; c Dept of Molecular Biology and Immunology, Faculty of Medicine, Hasanuddin University Makassar, Indonesia; d Dept of Physiology, Faculty of Medicine, Hasanuddin University Makassar, Indonesia; e Dept of Pulmonology, Faculty of Medicine, Hasanuddin University Makassar, Indonesia; f Dept of Neurosurgery, Faculty of Medicine, Hasanuddin University Makassar, Indonesia; g Dept of Biochemistry, Faculty of Medicine, Hasanuddin University Makassar, Indonesia; h Dept of Pediatric, Faculty of Medicine, Hasanuddin University Makassar, Indonesia; i Dept of Surgery, Faculty of Medicine, Hasanuddin University Makassar, Indonesia; j Bhakti Medicare Hospital, Sukabumi, West Java, Indonesia

**Keywords:** mRNA TLR2, TLR4, CD4, BCG scar, Tuberculosis, Children

## Abstract

**Background:**

How far the role of innate immunity and adaptive immunity do in children who have been BCG vaccinated in controlling the course and the severity of the TB disease has not been completely known. Mycobacterium tuberculosis entry to the body will be recognized by Toll-like receptors found on macrophages, neutrophils, and dendritic cells as part of the innate immune response, after which the dendritic cells will then present the antigen to lymphocyte T0 cells and initiate the adaptive immune response (of which CD4 T cells have an important role in). Was one or were both of these immune responses function well or not in a BCG Vaccinated Children with TB?

**Objective:**

This study aim to find a better understanding of the role of innate immune response assessed by TLR2/TLR4 mRNA gene expression and serum TLR2/TLR4 levels, while the role of adaptive immune response is assessed by analyzing serum CD4 level in children with TB who have had BCG vaccination.

**Methods:**

This cross-sectional study was conducted among children with TB at the outpatient and inpatient wards at Bhakti Medicare and Jakarta Islamic Hospital. Expression of mRNA gene was measured using the Boom method and protein serum levels were measured using the ELISA method. The results were analyzed by using the SPSS v.23 program.

**Results:**

Sixty-nine children were recruited as subjects. In this study, 68.1% of whom had BCG scars. TLR4 mRNA gene expression was found to be higher than TLR2 mRNA gene expression. Serum CD4 level was found to be highest out of TLR2 and TLR4 level, but serum TLR2 level was higher than TLR4 level. TLR2/TLR4 mRNA gene expression, serum TLR2/TLR4 levels, and CD4 levels in subjects with BCG scar were also found to be significantly higher than in subjects without BCG scar (p < 0.001). There was a significant positive correlation between TLR2/TLR4 mRNA gene expression and serum TLR2/TLR4 levels (r = 0.860; r = 0.864; p < 0.001) and between serum levels TLR2/TLR4 with serum CD4 levels (r = 0.822; r = 0.832 p < 0.001).

**Conclusion:**

As early as possible, BCG vaccine administration is needed in endemic countries, but it must be ensured that scars can be formed. It is also important to control Latent TB Infection (LTBI) to prevent transmission and relapse of disease.

## Highlights

• For the prevention of TB disease, it is important to understand the innate and adaptive immune responses according BCG scar.
• Mycobacterium tuberculosis recognition by TLR as the innate immune response followed by the adaptive immune response by CD4 cells.
• There is a strong positive correlation between the expression of mRNA gene and the concentration of TLR and CD4 in BCG-vaccinated subjects.
• BCG vaccination should be done as early as possible in endemic countries, as it provides better protection against TB infection.

## 1 Introduction

Indonesia is ranked second worldwide in terms of estimated tuberculosis (TB) incidence rate per year [1]. Some of the problems that cause TB disease cannot be controlled, including drug resistant TB caused by host factor that create susceptibility to Mycobacterium infection, various strains of Mycobacterium isolates and the form of anti-tuberculosis drug preparation (FDC) [[2], [3], [4], [5], [6]]. Another problems is there is still limited facilities for the purpose of TB diagnosis, such as the rapid molecular examination (GenXpert examination) [[7], [8], [9]].

Another reason of concern is the protective ability of the Bacille Calmette-Guérin (BCG) vaccine in high endemic areas like Indonesia, where immunization coverages is still lacking (90%) [10]. BCG vaccination is still one of the recommendations given by WHO to prevent *Mycobacterium tuberculosis* (MTB) infection. BCG scar formation following vaccination indicates a well functioning immune system [11,12].

The activation of innate host defense begins with the pattern recognition of the pathogen by specific pattern recognition receptors (PRR) bearing cells which then trigger the production of proinflammatory cytokines and chemokines that will initiate phagocytosis to kill *Mycobacteria* (MTB) [13,14]. Pattern Recognition Receptors such as Toll Like Receptors (TLRs), Nod-like receptors (NLRs) and C-type lectin receptors (CLRs) which detect distinct evolutionarily conserved structures on pathogens, termed pathogen-associated molecular patterns (PAMPs) from MTB (such as glycolipids, lipoproteins and carbohydrates) and cellular injury known as Damage-associated molecular patterns (DAMPs) [13,14]. DAMP are endogenous danger molecules that are released from damaged or dying cells from both infectious and sterile insults, they promote pathological inflammatory responses leading to chronic inflammation, mediate tissue repair gene synthesis and cause TLR2-dependent DC maturation [15,16]. Previous reports showed that serum levels of TLR2 and TLR4 Extrapulmonary TB (EPTB) were more higher than Pulmonary TB (PTB), that was subjects with EPTB showed be associated severe clinical symptoms, with broad tissue damage that indicated a greater innate immune response in EPTB [17].

Toll-like receptors (TLRs) are a family of PRRs consisting of 12 members in mammals, which one involved in recognition of MTB are TLR2, TLR4, TLR9, and possibly TLR8 [14,18]. TLR2 is important in the initiation of innate host defense through its stimulatory effects on the secretion of chemokines and cytokines, mainly tumor necrosis factor-*α* (TNF*α*), cytokines of the interleukin-1 family (IL-1*β*, IL-18), IL-12, nitric oxide and interferon-*γ* (IFN*γ)*. TLR2 is effective in inducing granuloma formation and controlling chronic infection with MTB and TLR4-induced activation of the phagosome-lysosome fusion [14]. Multiple cell types of the adaptive immune system respond to MTB infection, but CD4 T cells are the principal antigen-specific cells, and the major contributors to the disease in both host protection and immunopathology during MTB infection. Dendritic cells (DC) will present the antigen to initiation adaptive immunity response [13,14,19].

There are many unknown details about the immune response as well as the biological events that occur in the early stages of exposure and during the occurrence of infection and disease [20]. The innate immune system is the first line of defense against to all germs and foreign substances, that it was called “nonspecific” immune system. Upon recognition of PAMP and DAMP by cell surface membrane and intracellular TLRs, adaptor proteins containing TIR domains will be recruited. This will initiate signal transduction pathway that culminates in expression. Finnaly will activated complement pathways, stimulate chemokine and pro-inflammatory cytokine production and effectivitas opsonization and phagocytosis to clear or control the infection [13,14]. Failure or insufficient of the innate immune response will cause the mycobacteria can invade the lung parenchyma. Adaptive immune responses are triggered when macrophages and dendritic cells present MTB antigens to T-cells, including Th-1 type CD4^+^ T-cells, CD8^+^ cytotoxic T-cells, and gamma-delta (γδ) T-cells which further potentiate key cytokine secretion for MTB control [13,14,20].

This study aimed to identify the immunology respon from relationship between the innate immune response as assessed by the expression mRNA TLR2 and TLR4, serum levels of TLR2 and TLR4 with the adaptive immune response assessed from serum level of CD4 in TB children have vaccinated.

## 2 Materials and methods

The study was conducted among children with TB in Bhakti Medical Sukabumi and Jakarta Islamic Hospital between November 2018 to December 2019. A pediatrician established the diagnosis of pulmonary tuberculosis (PTB) and extrapulmonary tuberculosis (EPTB) according to clinical symptoms, physical examination, hematology and radiology examination or ultrasonografi or CTscan. Tuberkulin Skin Test (TST) was done using 0,1 mL of Tuberculin PPD RT 23-2TU (Purified Protein Derivative, with 2 Tuberculin Units) which was injected intradermally into the volar of the forearm and after 48–72 hours the diameter of induration formed were measured, the result is positive if the diameter ≥10 mm.

Vaccination status were assessed by way of anamnesis or looking at the vaccination card, and the right upper arm of the subject was then inspected for a BCG scar. The inclusion criteria children 0–18 years old registered patient who have been vaccinated BCG. The exclusion criteria are children with heart disorder, brain abnormalities, autoimmune diseases, congenital abnormalities, diabetes mellitus, renal disease, and received immunosupressant drugs and those who were consuming anti TB agent for more than 2 weeks. The children were included in this study after their parents signed informed consent.

### 2.1 Ethics statement

The Health Research Ethics Committee of the Hasanuddin University Hospital of Medicine, Indonesia, approved of this study (371/UN4.6.4.5.31/PP36/2019 on May 15, 2019). In this study, the authors confirmed that all methods were carried out under the relevant guidelines and regulations (Helsinki Declaration) under the number researchregistry7497.

### 2.2 Measuring expression of mRNA gene TLRs and serum of TLR and CD4 levels

Two ml of peripheral blood was collected under sterile conditions and divided into two samples; one sample was for Real-time polymerase chain reaction (RT PCR) used to measure TLR2/TLR4 mRNA gene expression and the other for ELISA to measure serum TLR2, TLR4 and CD4 levels. The samples were immediately centrifuged at the speed of 3000 rpm for 10–15 minutes to separate blood cells and serum. The serum was placed into an Eppendorf tube and stored in a freezer at −20 °C pending their analysis. The samples were coded and RT PCR/ELISA was performed without prior knowledge of the classification of the samples. The samples would be sent to the Laboratory of the Medical Faculty of Hasanuddin University Makassar, Indonesia for PCR/ELISA analysis.

### 2.3 Nucleic acid extraction

A sample volume of approximately 100 μL of blood was added to 900 μl of “L6” solution consisting of 120 g Guanidium thyocianate (GuSCN) (Fluka Chemie AG, Buchs, Switzerland, cat no. 50990) in 100 mL of 0.1 M Tris HCl pH 6.4, 22 mL 0.2 M Ethylene Diamine Tetra Acetate (EDTA) pH 8.0, and 2.6 g Triton X-100 (Packard, Instruments), with a final concentration of 50 mM Tris HCl, 5 M GuSCN, 20 mM EDTA, and 0.1% Triton X-100. The result was then heated in a water bath at a temperature of 56 C for 10 minutes and added to 60 μl of “TE” solution consisting of 1 mM EDTA in 10 mM Tris HCL pH 8.0, then vortexed and centrifuged at a speed of 13.000 rpm for 2 minutes. The next process was incubating the liquid in oven for 10 minutes at 56 C. After incubation, vortex and re-centrifuge the liquid for 30 seconds at a speed of 13.000 rpm and the supernatant was taken. The nucleotide extract will be obtained from the supernatant in this process and stored at −80 C before PCR analysis [21].

### 2.4 TLR2, TLR4, dan β-actin primers for expression mRNA gen TLR2, dan TLR4 (Macrogen, Korea)

TLR2 F:ATTGTGCCCATTGCTCTTTC and TLR2 R:CTGCCCTTGCAGATACCATT;

TLR4 F:CCGCTTTCACTTCCTCTCAC and TLR4 R:CATCCTGGCATCATCCTCAC;

β-Actin F:CAAGATCATTGCTCCTCCTG and β-actin R:ATCCACATCTGCTGGAAGG [22].

### 2.5 Determine mRNA expression profile of TLR2 and TLR4 genes by *Real Time polymerase chain reaction* (RT-PCR)

Process of primary oligonucleotide-specific genes for TLR2, TLR4, and β-actin genes (Macrogen, Korea) are used as housekeeping genes (internal control). PCR condition was set at a temperature of 95 °C for 10 seconds and 57 °C for 30 seconds for 40 cycles. The condition was adapted according to a previous research protocol where qrt-PCr was used the sybrgreen qRT-PCR master mix kit, one step (Qiagen, USA). This protocol is optimized for the CFX Connect System (USA) real-time PCR machine instrument. The PCR program is then ready to run on a Realtime PCR machine (CFX Connect system, Biorad Laboratories, Real Time PCR 96 well 0.1 mL, USA). The value of ΔCt will be determined by subtracting the target Ct of each sample from the Ct value of its ß –actin [21,[23], [24], [25]]**.**

### 2.6 ELISA

First, 100 μL of diluent assay containing a protein buffer was added to each well. Subsequently, 100 μL of standardized fluid containing recombinant human serum of TLR2, TLR4, and CD4 from a specific kit was added. (Human TLR2 ELISA Kit Catalog No: LS-F12773; Human TLR4 ELISA Kit Catalog No: LS-F22086, and Human CD4 ELISA Kit Catalog No: LS-F6263, LSBio, USA) The samples were incubated for 2 hours at room temperature and the liquid in each well was rinsed with sterile phosphate buffered saline (PBS) four times in succession. Furthermore, 200 μL of horseradish peroxidase (HRP) conjugated streptavidin was added to each well, covered with aluminium foil, and incubated for 30 minutes at room temperature in the dark. The liquid was rinsed four times using sterile PBS. Next, 200 μL of substrate solution containing 3,3′,5,5′-tetramethylbenzidin (TMB) was added to each well, and the samples were incubated for 20 minutes at room temperature in the dark. The reaction was them stopped by adding 50 μL of stopping solution containing H_2_SO_4_ to each well and the samples were read using ELISA Reader 270 (Biomerieux, France) with a wavelength of 450 nm in 30 minutes. The concentrations of TLR2, TLR4, and CD4 were recorded in units of ng/mL [23].

### 2.7 Statistical analysis

The design of the study is cross-sectional with subjects recruited through a consecutive sampling methods. Statistical analysis was done using the SPSS v.23 program, with descriptive analysis and bivariate analysis using *chi-square* test*,* and normality test of numerical data with Kolmogorof Smirnov, *independent t-test*, and *Pearson Correlation* test*.* Statistical tests were significant if p < 0.05.

## 3 Results

### 3.1 Characteristics demographic subject

In this study, 69 subjects with TB were vaccinated by BCG, 47 subjects (68,1%) of whom had BCG scars, while 22 subjects (31,9%) had no BCG scars, 36 subject (52.2%) were male, 38 subjects (55.1%) were under 6 years, 39 subjects (56.5%) were malnourished, and 35 subjects (50,7%) had no history of TB contacts. The results of the diagnostic examination using the tuberculin skin test (TST) were positive in 38 subjects (55,1%) and negative in 31 subjects (44.9%) (Table 1).

**Table 1:** Characteristic demographic subject.

Characteristic		N	%
Gender	Male	36	52,2
	Female	33	47,8
Age	0–6 years	38	55,1
	6–12 years	22	31,9
	13–18 years	9	13,0
Nutrition status	Malnutrition	39	56,5
	Wellnourish	30	43,5
History of contact TB	Positive	34	49,3
	Negative	35	50,7
BCG Scar	Positve	47	68,1
	Negative	22	31,9
TST	Positive	38	55,1
	Negative	31	44,9

∗descriptive statistic.

### 3.2 Characteristics of the subjects based on BCG scar status

In this study, 69 subjects with TB were vaccinated, 47 subjects (68,1%) of whom had BCG scars while 22 subjects (31,9%) had no scars. In children with BCG scar, 25 subjects (75.8%) were female, and most of them were under 6 years 28 subjects (73,6%), 26 subjects (66,7%) were malnourished, and 23 subjects (65,7%) had no history of TB contacts. The results of the diagnostic examination using the tuberculin skin test (TST) were positive in 27 subjects (71,1%) and negative in 20 subjects (64,5%). The results of statistical analysis with chi-square showed that all characteristics did not have a significant difference with BCG scar status (p > 0,05). From the interview results, no severe or moderate disease was found in children under 6 years, and the subjects came from lower to middle socioeconomic status (Table 2).

**Table 2:** Characteristics Subjects based on Scar BCG Status.

Characteristics		Total (n = 69)	BCG scar positive (n = 47)	% 68,1	BCG scar negative (n = 22)	% 31,9	p-Value
Gender	Female	33	25	75,8	8	24,2	0,192
	Male	36	22	33,3	14	66,7	
Age	<6 years	38	28	73,6	10	26,3	0,768
	6–18 years	31	19	61,2	12	38,7	
Nutritional Status	Undernutrition	39	26	66,7	13	33,3	0,272
	Normal	30	21	70,0	9	30,0	
History of	None	35	23	65,7	12	34,3	0,664
TB contact	Yes	34	24	70,6	10	29,4	
Tuberculin skin Test	Negative	31	20	64,5	11	35,5	0,562
	Positive	38	27	71,1	11	28,9	

∗chi-square test.

### 3.3 TLR2/TLR4 mRNA gene expression and serum levels of TLR2/TLR4 and CD4

Based on the research results, the mean TLR2 mRNA gene expression 9,714 fc was lower than mean TLR4 mRNA gene expression 11,640 fc and the mean serum CD4 level 63,790 ± 16,628 SD were higher than serum TLR2 (26,225 ± 12,394SD) and TLR4 levels (13,460 ± 3,938,SD) respectively. This table showed serum TLR2 level were higher than serum TLR4 level. There were no significant differences found of TLR2/TLR4 mRNA expression serum CD4 levels, serum TLR2 level, and serum of TLR4 based on age classification, gender, nutrition status, and history of contact with TB (data not shown) (Table 3).

**Table 3:** Expression of mRNA and serum levels of TLR2, TLR4, and CD4.

	n	Mean (SD)	Min -max^a^	Median
Expression of mRNA				
TLR2 (fc)	69	9,714 (2,160)	5,137–12,996	10,032
TLR4 (fc)	69	11,640 (2,173)	7,091–14,920	12,154
**Serum Levels**				
TLR2 (ng/mL)	69	26,225(12,394)	2,822–48,404	26,271
TLR4 (ng/mL)	69	13,460 (3,938)	6,003–20,379	13,602
CD4 (ng/mL)	69	63,790(16,628)	30,658–93,878	63,857

a Descriptive statistic.

### 3.4 TLR2/TLR4 mRNA gene expression and serum level of TLR2, TLR4, and CD4 based on clinical manifestation status

Based on the clinical manifestation, the average expression of mRNA gene TLR2 and expression of mRNA gene TLR4 was higher in severe clinical manifestation than mild and moderat clinical manifestation with significantly different (11,68 fc VS 9,55 fc and 13,58 fc VS 11,48 fc (p < 0,05)) respectively. But the expression of mRNA gene TLR4 more higher than expression of mRNA gene TLR2 (13,58 fc VS 11,68 fc) respectively. There are showed the average serum levels of CD4 were highest than TLR2 and TLR4, and serum levels of TLR2 more higher than serum levels of TLR4 (80,37 (ng/mL); 34,49 (ng/mL); 16,96 (ng/mL) respectively. Based on clinical manifestation that shows serum levels of CD4 and TLR4 were higher in severe clinical manifestation than mild and moderat clinical manifestation with significantly different (80,37 ng/mL VS 60,80 ng/mL and 16,96 ng/mL VS 13,18 ng/mL (p < 0,05) respectively), but for serum levels of TLR2 there are no significant difference (34,49 ng/mL VS 25, 57 ng/mL (p > 0,05) although serum level of TLR2 more higher than TLR4 (Table 4).

**Table 4:** Expression of mRNA and Serum Levels of TLR2, TLR4, and CD4 based on Clinical Manifestation Status.

		Clinical Manifestation		p-value
		Severe (n = 5)	Mild Moderate (n = 64)	
Expression of mRNA				
TLR2 (fold change)	Mean(SD) 95% CI	11,68(1,20) 10,18–13,17	9,55(2,14) 9,02–10,09	**0,033**
TLR4 (fold change)	Mean(SD) 95% CI	13,58(1,53) 11,66–15,49	11,48(2,14) 10,95–12,02	**0,037**
**Serum Levels**				
TLR2 (ng/mL)	Mean(SD) 95% CI	34,49(6,77) 26,08–42,90	25,57 (12,53) 22,44–28,70	0,122
TLR4 (ng/mL)	Mean(SD) 95% CI	16,96(2,57) 13,76–20,16	13,18 (3,90) 12,21–14,16	**0,038**
CD4 (ng/mL)	Mean(SD) 95% CI	80,37(12,55) 64,78–85,96	60,80 (17,02) 56,71-64,88	**0,014**

∗descriptive statistic.

**Fig. 1:**
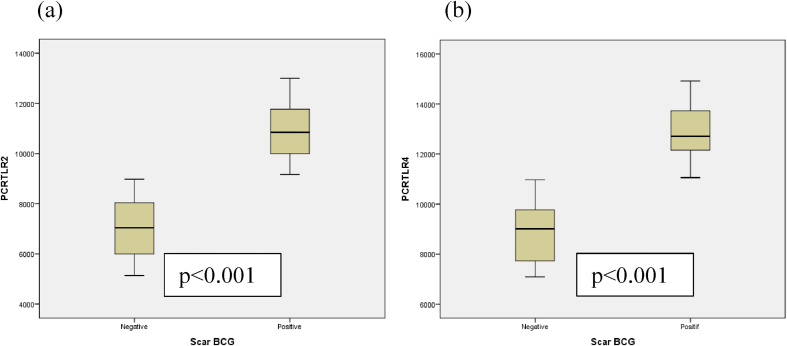
Showed box plot the comparison between expression of mRNA gene TLR2 (a) and expression of mRNA gene TLR4 (b) in subjects with scar positive show significant difference than in subjects scar negative (p < 0.001) and expression of mRNA gene TLR4 increases than the expression of mRNA gene TLR2. The boxes represent the interquartile range (25–75% of the sample); the line in the middle is the median (50%). The dots are outliers as the whiskers extend up to 1,5 times the interquartile range.

Table 5, it can be seen that the mean TLR2 mRNA gene expression in subject with BCG scars was 10,96 ± 1,10 SD compared to 7,03 ± 1,19 SD in subject without BCG scar. Analysis using independent T-Test showed a significant relationship between the mean TLR2 mRNA geneexpression with BCG scar status (p < 0,001). The mean TLR4 mRNA gene expression in subject with BCG scar was 12,89 ± 1,08 SD compared to 8,94 ± 1,25 SD in subject without BCG scar. Analysis using the independent T-Test showed a significant relationship between the mean TLR4 mRNA gene expression with BCG scar status (p < 0,001). The difference in the mean expression of mRNA TLR2 and TLR4 genes against the BCG scar is visible in (Fig. 1).

**Table 5:** Expression of mRNA Gene TLR2 and TLR4 based on BCG Scar.

Expression of mRNA Gene	Scar BCG Positive		Scar BCG negative		p-value
	Mean (SD)	95% CI	Mean (SD)	95% CI	
TRL2 (fc)	10,96 (1,10)	10,64–11,29	7,03 (1,19)	6,51–7,56	<0,001
TRL4 (fc)	12,89 (1,08)	12,58–13,21	8,94 (1,25)	8,39–9,50	<0,001

∗independent *t*-test.

**Fig. 2:**
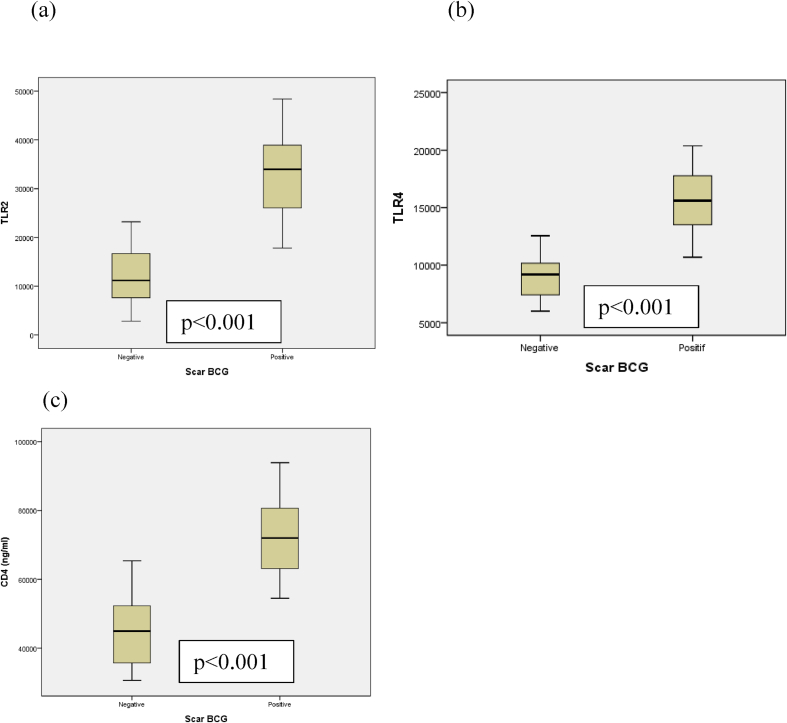
Showed box plot comparison between serum level of TLR2 (a) and serum level of TLR4 (b) and serum level of CD4(c) in subjects with scar positive showed significant difference than subjects scar negative (p < 0.001) and serum level of CD4 were highest than TLR2 and TLR4. The boxes represent the interquartile range (25–75% of the sample); the line in the middle is the median (50%). The dots are outliers as the whiskers extend up to 1,5 times the interquartile range.

Table 6 showed that the mean serum level of TLR2 in subject with BCG scar was (32,78 ± 8,60 SD)compared to (12,21 ± 5,77 SD) in subject with no BCG scar. Mean serum level of TLR4 in subject with BCG scar was 15,54 ± 2,76 SD) compared to (7,03 ± 1,19 SD) in subject with on BCG scar. Mean serum level of CD4 in subject with BCG scar was (72,36 ± 11,11 SD) compared to (45,49 ± 10,47 SD) in subject with no BCG scar. Analysis using the independent T-Test showed a significant relationship between serum levels TLR2, TLR4, and CD4 with BCG scar status compred without BCG scar (p < 0,001). The difference in the mean serum TLR2, TLR4, and CD4 levels against the BCG scar is visible in Fig. 2.

**Table 6:** Serum Levels of TLR2, TLR4, and CD4 based on BCG Scar.

Serum Levels	Scar BCG Positive		Scar BCG negative		p-value
	Mean (SD)	95% CI	Mean (SD)	95% CI	
TRL2 (ng/mL)	32,78 (8,60)	30,25–35,30	12,21 (5,77)	9,66–14,77	<0,001
TRL4 (ng/mL)	15,54 (2,76)	14,72–16,35	7,03 (1,19)	8,24–9,78	<0,001
CD4 (ng/mL)	72,36(11,11)	69,09–75,62	45,49(10,47)	40,84-50,13	<0.001

∗independent *t*-test.

### 3.5 Correlation between expression of mRNA gene TLR2/TLR4 with serum levels of TLR2/TLR4 and CD4

Table 7 shows based on pearson correlation test showed correlation TLR2 mRNA gene expression and serum TLR2 level, TLR2 mRNA gene expression with TLR4 mRNA gene expression, TLR2 serum level with TLR4 serum level, TLR2 serum level with serum CD4 level, and TLR4 mRNA gene expression with serum TLR4 level, serum TLR4 level with serum CD4 serum level were significantly different with r = 0.860, r = 0.933, r = 0.865, r = 0.822, r = 0.864, and r = 0,832 respectively (p < 0.001). The relationship between all examined variables showed a strong positive correlation.

**Table 7:** Correlation between expression of mRNA gene TLR2/TLR4 with serum of levels TLR2/TLR4 and CD4.

Correlation		Bivariate Analysis	
Variable 1	Variable 2	Coefficient	p-Value^a^
Expression of mRNA gene TLR2	Serum TLR2 level	r = 0,860	<0,001
Expression of mRNA gene TLR2	Expression of mRNA gene TLR4	r = 0,933	<0,001
TLR2 Serum level	TLR4 Serum level	r = 0,865	<0,001
TLR2 Serum level	CD4 Serum level	r = 0,822	<0,001
Expression of mRNA gene TLR4	TLR4 Serum level	r = 0,864	<0,001
TLR4 Serum level	CD4 Serum level	r = 0,832	<0,001

a Pearson correlation test.

The results showed that if the mean TLR2 mRNA gene expression is high, the serum TLR2 level will be higher. If the mean TLR2 mRNA gene expression is high, the TLR4 mRNA gene expression will be higher. If the mean serum TLR2 level is high, the TLR4 serum level will be even higher. If the mean serum TLR2 level is high, the serum CD4 level will be higher. and if the mean TLR4 mRNA gene expression is high, the serum CD4 level will also be higher.

## 4 Discussion

The protection of BCG vaccine can last up to 10–15 years. It may last longer when BCG is administered at birth, especially in low incidence country [[26], [27], [28]]. The vaccine can induce nonspecific cross-protection against pathogen-unrelated targeting disease and can decrease the mortality and morbidity of TB and several diseases in infants [12,29,30]. The BCG vaccine has been around for a long time. It was then distributed to many laboratories in the world. BCG subcultures were re-reproduced and led to the phenotypically different vaccine strains. Due to the difference in handling the storage, the effectiveness of the vaccine was not the same as the original [31,32]. Comparative genomic studies have documented that BCG vaccine strains were different from the original BCG which was first used in 1921. The genetic differences will affect the antigenic proteins and its effectiveness [31,33].

In this study, 69 children with extra and intratuberculosis who had been vaccinated by BCG were enrolled. In endemic areas, there is a high probability of TB infection, while the effectiveness of vaccine protection was only 42%–85%. The two factors can make even children who have been vaccinated with BCG can still contract tuberculosis [34]. There are no severe clinical manifestation in subject who are under five years old and we know scar formation was associated with reduced mortality and increased infant survival in the first and second years of life, especially if the vaccination was done in the neonatal period [11,30,35,36]. The protection's effication of vaccines affected by age at the time of administration, history of sensitization to environmental microbes, birth weight, gestational age at birth, injection technique, vaccinator's skill, strain/type of the vaccine, wheal occurred at the injection site, and scar formation on mother [11,34,37].

The scar formation is influenced by the type and strain of vaccine, age of administration, technique of injection, genetic factors, nutritional status, poor cold chain management, simultaneous oral polio vaccine administration, exposure to environmental Mycobacterium, gestational age at birth, and different subspecies [[38], [39], [40], [41]]. The subjects with scar are about 68.1%, most of them female, under 6 years old, with undernutrition, and part of them with a positive history of TB contact. There was no significant differences between the subject with or without scar according to categorical variable such as gender, age, nutrition state, history of TB contact, and TST examination. A study with a larger number of subjects and a more suitable study design is needed to understand a relationship between BCG scar formation with several factors, especially age and nutritional status. This study did not completely observe the management and administration of BCG vaccination in subjects.

Positive TST appeared in 55% subjects, mostly under 6 years old, and were still influenced by the effect by BCG. False negative might happen to other subjects due to anergy, overwhelming TB, or recent TB infection [42,43]. IGRA has higher specificity and negative predictive value than TST in children above 5 years old that had BCG vaccination. Unfortunately, IGRA was not available due to its high cost and it was not covered by publich health insurance [42,44].

The mechanism of Tuberculin Skin Test (TST) was that of a delayed (cellular) hypersensitivity reaction, when T cell were sensitized by prior infection with Environmental Mycobacterium (EM). Langerhan's cells will be processes the antigen and present antigen to local memory T cells, CD4^+^ or CD8^+^, and they will release cytokines (TNFα and IFN) and lymphokines as the early hallmarks of inflammation [43,45]. Local vasodilatation, edema, fibrin deposition, and recruitment of other inflammatory cells will be forming the induration. Tuberculin Skin Test does not measure immunity against TB but is used as assessment of the degree of hypersensitivity to tuberculin and to determine the prevalence of latent tuberculosis infection in population [23,43,45]. This study revealed the TLR2/TLR4 mRNA gene expression in subject with positive TST were significantly different than in subjects with negative TST (p < 0.001). The serum TLR2, TLR4, and CD4 levels also showed significant difference based on TST reaction (data not shown) [23].

TLR2 and TLR4 are cell surface TLRs which are expressed on immune cells, endothelial epithelial cells, and respiratory epithelial cells, that are important to host protection and immune evasion by MTB during acute and chronic infection [13,14,18]. Active and latent MTB infections can increase the TLR2 and TLR4 mRNA gene expression, which are important PRRs to recognize MTB and initiate effective innate immune response [14,18]. This study showed TLR4 mRNA gene expression was higher than TLR2 but conversely the amount serum level of TLR2 was higher than TLR4. Based on clinical manifestation that showed differently significant between TLR4 and TLR2 mRNA gene expression and serum levels of TLR4 and CD4 in severe clinical manifestation than mild moderate clinical manifestation, eventhough those who are seriously ill are only 5 subject and they are above 5 years old. Subject with severe manifestation can be leading to a greater immune response which one recognition of more DAMPs by TLR receptors and caused of extensive tissue damage [[15], [16], [17]].

The other reason the receptors of TLR2 have more ligands than TLR4. The known receptors are lipomannan, lipoarabinomannan, lipoprotein, lipotechoic acid and HMBG1 was found in Mycobacterium spescies, gram positive bacteria, viruses, and parasites [46]. The receptors TLR2 also forms a heterodimer with TLR1 and TLR6, which escalate the function of TLR2. Meanwhile receptors TLR4 also forms a heterodimer too with TLR1, but the bond only reduces TLR4 performance [18,47]. TLR2 heterodimers with TLR1/TLR6, are involved in a cascade of events leading to significant innate immune responses and amplified TLR4 signaling induces neutrophil chemotactic CXCL8 (interleukin 8) with activation of The ERK1/2 MAPK pathway to elimitation pathogens [18,48].

The involvement of genetic factors in TLR mRNA gene expression has been demonstrated in the form of single nucleotide polymorphisms (SNP), the TLR1 rs5743618-GT genotype. A decrease in TLR1 expression on the surface of monocytes and granulocytes is associated with child susceptibility to TB [49]. Several stages in the gene expression process are transcription, RNA splicing, translation, and post-translational modification of proteins. Gene regulation provides cell control of structure and function, and is the basis for cell differentiation, morphogenesis, and the versatility and adaptability of any organism. Gene regulation can also as a substrate for evolutionary change, as the control of the timing, location, and amount of gene expression can have major effects on gene function (action) in cells [50].

Others study reported increased TLR2/TLR4 mRNA gene expression in latent tuberculosis infection (LTBI) than in non infected, and increased in active TB than LTBI or healthy individuals [51,52]. This research reported the mean fold changes of TLR4 mRNA gene expression in children with active TB were increased than TLR2 mRNA gene expression. There was a significant difference between the expression of the TLR2/TLR4 mRNA gene expression based on the presence of scar (p < 0.001).

There were 64 subjek with mild manifestation and 5 subjects with severe clinical manifestation and according tuberculosis diagnose there were 57 subjek with PTB and 12 subjek with EPTB [17]. The active TB and LTBI can caused tissue damage as DAMP, and level of tissue damage depends on the presence and severity from clinical manifestation and duration of illness. The DAMP will activate innate immune response through TLR2, TLR4, TLR7, TLR8, and TLR9 receptors which are found in macrophages and others cells, and different pathways will trigger inflammatory responses including TLR and inflammasomes [[15], [16], [17]]. Involvement of the organs other than lungs in infection tuberculosis will be enhancement the innate and adaptive immune responses and show more severe symptoms.

BCG vaccination causes an immune activation of CD4 T cells but no activation of CD8 T cells in BCG-vaccinated and unvaccinated infants [53]. The enhancement of adaptive responses after BCG vaccination have reported through assessment with TST and the result was BCG scar size correlated with Th2 response early after vaccination and Th2 response decline over time [29]. The apoptotic neutrophils proccesed play an important role in the activation of MTB-specific CD4 T cells by modulating the migratory of dendritic cells [19]. Natural Killer (NK), CD4^+^, and CD8^+^ T cells secretion IFNγ after release of endogenous IL-12 and IL-18 by macrophages and dendritic cells [13,14]. IFNγ can activate macrophages to kill and eliminate mycobacteria and enhance their expression of MHC class II molecules, which results in improved antigen presentation to T cells [19]. The means of serum CD4 levels were highest than the TLR2 and TLR4 serum levels, respectively, there are significantly difference between subject with scar and without scar BCG (p < 0.001).

This study revealed that there was a very strong positive correlation between TLR2 mRNA gene expression and TLR4, with serum levels of TLR2, TLR4, and CD4 with significant results (p < 0.001). The correlation between TLR4 mRNA expression and serum TLR4 level different, that caused influenced by several factors from TLR2, TLR 4, the clinical manifestation of the subject and others. The coordination TLR2 and TLR4 dependent mediated signals play in macrophage apoptosis, and TLR4 is important in the maintenance of the balance between apoptotic vs. necrotic cell death [54]. The TLR2 triggered proinflammatory cytokines initiate protective mechanisms and limit MTB replication, while the immune evasion pathways counterattacks antibacterial effector mechanisms [55,56].

The intracellular pathogens in animals and human have extensive polyfunctional CD4^+^ T cells that may potentially play a role in vaccine-mediated protection including long-lived memory function, persistence in the vaccinated host, and the ability to traffic to and persist in the lung [57,58]. The polyfunctional CD4 T cells producing multiple proinflammatory cytokines (IFN-γ, TNF-α, and IL-2) which one correlate with protection from infection and disease. Mycobacteria-specific T-cell responses showed serum levels of IFN-γ, IL-2, were higher in children with TB than healthy dan HIV children [59]. In healthy children reveal an inverse correlation between age and the levels of the CD4 T cells response, several factors that may affect this, including the time of BCG vaccination and the natural waning of the proportion of naive T cells with age [[59], [60], [61], [62]].

MTB can also hijack TLR2 signaling to subvert host immunity by dampening the macrophages response to IFN-γ, suppressing antigen processing and presentation. These functional responses cause MTB persistence with minimal immunopathology [55,56].

### 4.1 Study limitation

The limitations of this study were the diagnosis of TB without molecular testing (Gen X-Pert), we did not analyze the timing of age at vaccination (days or month), did not analyze how close contact was with adult TB patients and did not involve healthy children who had been vaccinated. with BCG as a comparison of the studied biomarker values.

## 5 Conclusions

The optimal innate immune response plays an important role in the initiation of the adaptive immune response to control and defend against MTB infection, especially in children with BCG scars. BCG vaccine administration as early as possible is needed in endemic countries, but it must be ensured that scars can formed. It is also importance to controlling LTBI to prevent transmission to older children.

## 6 Suggestion

Further research is needed regarding the administration of BCG vaccine boosters for children in whom BCG scar were not formed or in school age children. Furthermore, choosing a good quality vaccine that ensures BCG scar formation is also important.

## Data availability

The data will be available on request through a data access committeae, institutional review board, or the authors themselves.

## Ethical approval

The Health Research Ethics Committee of the Hasanuddin University Hospital of Medicine, Indonesia, approved of this study (371/UN4.6.4.5.31/PP36/2019 on May 15, 2019).

## Sources of funding

Universitas Muhammadiyah Jakarta.

## Author contribution

1. Conceptualization funding acquisition role/writing-original draft: Rahmini Shabariah.
2. Conceptualization, role/writing- original draft: Mochammad Hatta, Ilhamjaya Patellongi.
3. Resources, project administration: Farsida.
4. Data curation, methodology: Irfan Idris, Arif Santoso, Andi Asadul Islam, Rosdiana Natzir.
5. Supervision: Bob Wahyudin, Warsinggih, Sanjoyo.
6. Formal analysis, validation: Rahmini Shabariah, Farsida.

## Consent

This study has received approval from the ethics committee and written informed consent was required for each participant.

## Trial registry number

1. Name of the registry: Correlation Expression of mRNA Gene TLR2/TLR4 with Serum level TLR2/TLR4 and CD4 in TB Children with BCG Scar: Cross-Sectional Study
2. Unique Identifying number or registration ID: researchregistry7497
3. Hyperlink to your specific registration (must be publicly accessible and will be checked): https://www.researchregistry.com/browse-the-registry#home/registrationdetails/61cd917a75c1da001e935ecb/

## Guarantor

Dr. Rahmini Shabariah, Sp.A.

## Declaration of competing interest

The authors declare that they have no competing interests.
